# 2,2,9-Trimethyl-2,3-dihydro­pyrano[2,3-*a*]carbazol-4-(11*H*)-one

**DOI:** 10.1107/S1600536808033850

**Published:** 2008-10-22

**Authors:** Makuteswaran Sridharan, Karnam J. Rajendra Prasad, Matthias Zeller

**Affiliations:** a Department of Chemistry, Bharathiar University, Coimbatore 641 046, Tamil Nadu, India; b Department of Chemistry, Youngstown State University, One University Plaza, Youngstown, OH 44555, USA

## Abstract

The title compound, C_18_H_17_NO_2_, was prepared from 1-hydr­oxy-7-methyl­carbazole and 3,3-dimethyl­acrylic acid with trifluoro­acetic acid as the cyclization catalyst. The mol­ecules contain an essentially planar 6-methyl­indole unit. The second aromatic ring is significantly bent away from the plane of this unit, with maximum deviations of 0.171 (1) and 0.185 (1) Å for two of the C atoms. In the crystal structure, there are neither N—H⋯O hydrogen bonds nor π–π stacking between the aromatic sections of neighboring mol­ecules. There is only one weak C—H⋯O hydrogen bond and a number of weak C—H⋯π inter­actions.

## Related literature

Knölker & Reddy (2002 ▶) report on the isolation of pyran­o­carbazoles from various plant species. Sridharan *et al.* (2007 ▶) describe the synthesis of compounds related to the title compound. Sridharan *et al.* (2008*a* ▶,*b* ▶) report the structures of the 9-*H* and 10-methyl derivatives of the title compound.
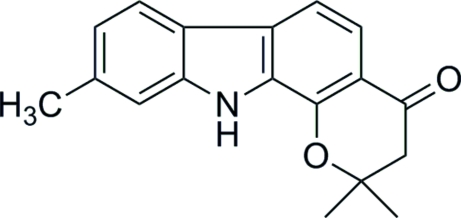


## Experimental

### 

#### Crystal data

C_18_H_17_NO_2_
*M* *_r_* = 279.33
Monoclinic, 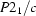

*a* = 8.6702 (7) Å
*b* = 6.1647 (5) Å
*c* = 26.617 (2) Å
β = 99.100 (1)°
*V* = 1404.7 (2) Å^3^
*Z* = 4
Mo *K*α radiation
μ = 0.09 mm^−1^
*T* = 100 (2) K
0.57 × 0.48 × 0.24 mm

#### Data collection

Bruker APEXII CCD diffractometer
Absorption correction: multi-scan (*SADABS*; Bruker, 2007 ▶) *T*_min_ = 0.883, *T*_max_ = 0.980
12327 measured reflections
3044 independent reflections
2708 reflections with *I* > 2σ(*I*)
*R*_int_ = 0.031

#### Refinement

*R*[*F* ^2^ > 2σ(*F* ^2^)] = 0.039
*wR*(*F* ^2^) = 0.098
*S* = 1.04
3044 reflections
193 parameters
H-atom parameters constrained
Δρ_max_ = 0.27 e Å^−3^
Δρ_min_ = −0.21 e Å^−3^

### 

Data collection: *APEX2* (Bruker, 2007 ▶); cell refinement: *SAINT* (Bruker, 2007 ▶); data reduction: *SAINT*; program(s) used to solve structure: *SHELXTL* (Sheldrick, 2008 ▶); program(s) used to refine structure: *SHELXTL*; molecular graphics: *Mercury* (Macrae *et al.*, 2006 ▶); software used to prepare material for publication: *SHELXTL*.

## Supplementary Material

Crystal structure: contains datablocks global, I. DOI: 10.1107/S1600536808033850/hb2804sup1.cif

Structure factors: contains datablocks I. DOI: 10.1107/S1600536808033850/hb2804Isup2.hkl

Additional supplementary materials:  crystallographic information; 3D view; checkCIF report

## Figures and Tables

**Table 1:** Hydrogen-bond geometry (Å, °)

*D*—H⋯*A*	*D*—H	H⋯*A*	*D*⋯*A*	*D*—H⋯*A*
C15—H15*B*⋯O2^i^	0.99	2.48	3.4034 (13)	155
N1—H1⋯*Cg*2^ii^	0.88	2.97	3.5822 (11)	128
C2—H2⋯*Cg*1^ii^	0.95	2.74	3.5332 (13)	141
C8—H8⋯*Cg*1^iii^	0.95	2.91	3.4124 (12)	114
C9—H9⋯*Cg*2^iii^	0.95	2.74	3.4372 (13)	130

Symmetry codes: (i) 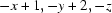; (ii) 
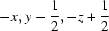
; (iii) 
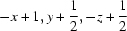
. *Cg*1 and *Cg*2 are the centroids of the N1/C1/C6/C7C12 and C1–C6 rings, respectively.

**Fig. 1.**
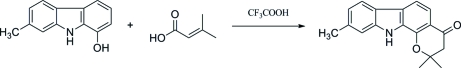
Reaction sequence

**Fig. 2.**
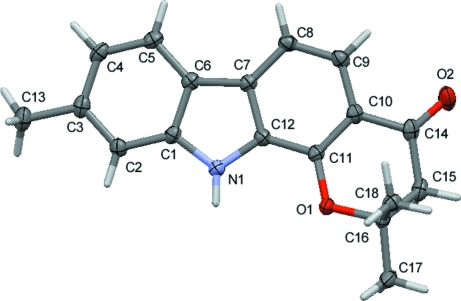
The molecular structure of (I) showing xx% displacement ellipsoids. H atoms are represented in stick mode.

**Fig. 3.**
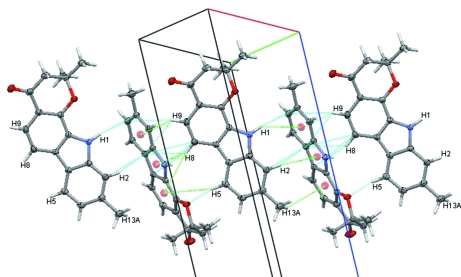
Packing view of (I) showing part of one of the layers held together by C—H···π interactions. Blue dashed lines indicate short N/C—H···C contacts, green dashed lines indicate N/C—H contacts towards centers of aromatic rings as defined in Table 1.

**Fig. 4.**
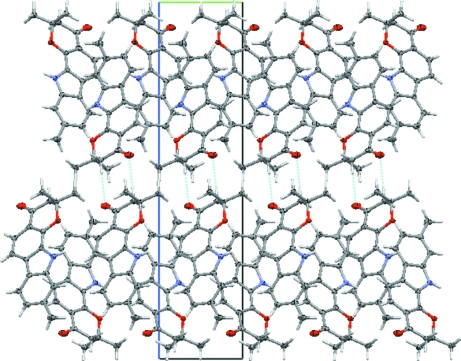
Packing view showing two of the layers held together by C—H···π interactions and the C—H···O hydrogen bonds between the layers. Blue dashed lines indicate short C—H···O contacts

## supplementary crystallographic information

### Comment

Carbazole alkaloids have been isolated from the taxonomically related higher plants of the genus Murraya, Glycosmis, and Clausena from the family Rutaceae. Among the carbazole alkaloids pyranocarbazole alkaloids play a very important role. In this class girinimbine was the first member of the pyrano[3,2-*a*]carbazole alkaloid family to be isolated from *M*. Koenigii Spreng (Knölker & Reddy, 2002, and references therein). The isolation of these classes of compounds became an active area of study since these compounds possess high levels of biological and pharmacological activity. Hence we attempted to synthesize pyranocarbazoles by a simple and efficient route.

Using trifluoroacetic acid as the acylating agent we had been able to synthezize in high yields a range of pyranocarbazolones and we recently reported (Sridharan *et al.*, 2007) the synthesis and crystallographic behaviour of 2,3-dihydro-2,2,8-trimethylpyrano[2,3-*a*]carbazol-4-(11*H*)-one. As an extension of this reasearch, and to further proof the credibility of trifluoroacetic acid as a good acylating agent, we further extended this synthetic route with a series of substituted 1-hydroxycarbazoles. The components thus synthesized were used as starting synthons to develop routes towards substituted pyranocarbazole derivatives.  Herein we report the crystal structures of the title compound, (I).

The molecules in (I) consist of an essential planar 6-methyl-indole unit made up of the atoms C1 to C7, C12 and N1. Also in plane with this unit are the atoms C8, C11 and O1, and the overall r.m.s. deviation from planarity for these atoms is 0.0319 Å. In the structures of two related compounds (Sridharan *et al.*, 2008*a* and 2008*b*) differentiated from the title compound only by the presence and/or position of one methyl group, the molecules were essentially planar with the only exception being the atoms of the C(CH_3_)_2_ unit of the pyranone rings. For the title compound, however, the remainder of the molecule, including the atoms C9 and C10 of the second aromatic six membered ring, are all deviating from the plane formed by the indole subunit. The aromatic ring is signifcantely bent away from this plane with C9 and C10 being displaced by 0.171 (1) and 0.185 (1) Å, respectively.

Differences between (I) and the two related structures (Sridharan *et al.*, 2008*a* and 2008*b*) extend into the crystal packing as well. The other compounds are dominated by strong N—H···O hydrogen bonds and also exhibit a range of π–π stacking as well as weak C—H···O interactions. The title compound, however, while only being differentiated from the other two molecules by the presence and/or position of one methyl group, does not exhibit any strong N—H···O hydrogen bonds and it also does not exhibit any π–π stacking between the aromatic sections of neighboring molecules. There is only one weak C—H···O hydrogen bond (Table 1). Other intermolecular interactions are limited to even weaker C—H···π interactions (Table 1, Figure 3). Centroids given in Table 1 are defined as follows: *Cg*1: the pyrrol ring (N1, C1, C6, C7, C12); *Cg*2: C1 to C6; *Cg*3: C7 to C12

In the absence of stronger interactions the large number of these contacts dominates the packing forces. Molecules within the unit cell are rougly aligned along the long axis of the unit cell (the *c* axis) and neighboring molecules are twisted against each other so that their planes are approximately perpendicular to each other, thus allowing the C—H···π interactions to establish themselves as shown in Figure 3. Each molecule is both C—H donor towards two neighboring molecules and C—H acceptor for two other neighbors. In combination this creates layers of molecules connected by C—H···π interactions. Each layer is in turn connected with parallel layers by weak C—H···O hydrogen bonds as shown in Figure 4.

### Experimental

1-hydroxy-7-methylcarbazole (0.001 mol) dissolved in 10 ml of trifluoroaceticacid was heated with 3,3-dimethylacrylicacid (0.001 mol) at 323 K for 5 h. The reaction was monitored by TLC. After completion of the reaction, the excess trifluroaceticacid was removed using rotary evaporation. The solid that precipitated out was poured onto ice water, then extracted using ethyl acetate and dried over anhydrous sodium sulfate and filtered. Then the solvent was removed under vacuum and the residue was purified by column chromatography on silica gel using petroleum ether/ ethyl acetate (95:5 v/v) as the eluant. Evaporation of solvent afforded yellow crystals which were recrystallized from ethanol to yield yellow blocks of (I) (0.262 g, 94%), m.p. 463–465 K.

### Refinement

All hydrogen atoms were added in calculated positions with C—H bond distances of 0.99 (methylene), 0.95 (aromatic) and 0.98 Å (methyl) and an N—H distance of 0.88 Å. They were refined as riding with U_iso_(H) = 1.2U_eq_(C,N) or 1.5U_eq_(methyl C).

### Crystal data

**Table:**

C_18_H_17_NO_2_	*F*(000) = 592
*M**_r_* = 279.33	*D*_x_ = 1.321 Mg m^−^^3^
Monoclinic, *P*2_1_/*c*	Mo *K*α radiation, λ = 0.71073 Å
Hall symbol: -P 2ybc	Cell parameters from 5407 reflections
*a* = 8.6702 (7) Å	θ = 2.4–27.0°
*b* = 6.1647 (5) Å	µ = 0.09 mm^−^^1^
*c* = 26.617 (2) Å	*T* = 100 K
β = 99.100 (1)°	Block, yellow
*V* = 1404.7 (2) Å^3^	0.57 × 0.48 × 0.24 mm
*Z* = 4	

### Data collection

**Table:**

Bruker APEXII CCD diffractometer	3044 independent reflections
Radiation source: fine-focus sealed tube	2708 reflections with *I* > 2σ(*I*)
graphite	*R*_int_ = 0.031
ω scans	θ_max_ = 27.0°, θ_min_ = 2.4°
Absorption correction: multi-scan (SADABS; Bruker, 2007)	*h* = −10→11
*T*_min_ = 0.883, *T*_max_ = 0.980	*k* = −7→7
12327 measured reflections	*l* = −33→33

### Refinement

**Table:**

Refinement on *F*^2^	Primary atom site location: structure-invariant direct methods
Least-squares matrix: full	Secondary atom site location: difference Fourier map
*R*[*F*^2^ > 2σ(*F*^2^)] = 0.039	Hydrogen site location: inferred from neighbouring sites
*wR*(*F*^2^) = 0.098	H-atom parameters constrained
*S* = 1.04	*w* = 1/[σ^2^(*F*_o_^2^) + (0.0431*P*)^2^ + 0.5637*P*] where *P* = (*F*_o_^2^ + 2*F*_c_^2^)/3
3044 reflections	(Δ/σ)_max_ < 0.001
193 parameters	Δρ_max_ = 0.27 e Å^−^^3^
0 restraints	Δρ_min_ = −0.21 e Å^−^^3^

### Special details

**Table:**

Geometry. All e.s.d.'s (except the e.s.d. in the dihedral angle between two l.s. planes) are estimated using the full covariance matrix. The cell e.s.d.'s are taken into account individually in the estimation of e.s.d.'s in distances, angles and torsion angles; correlations between e.s.d.'s in cell parameters are only used when they are defined by crystal symmetry. An approximate (isotropic) treatment of cell e.s.d.'s is used for estimating e.s.d.'s involving l.s. planes.
Refinement. Refinement of *F*^2^ against ALL reflections. The weighted *R*-factor *wR* and goodness of fit *S* are based on *F*^2^, conventional *R*-factors *R* are based on *F*, with *F* set to zero for negative *F*^2^. The threshold expression of *F*^2^ > σ(*F*^2^) is used only for calculating *R*-factors(gt) *etc*. and is not relevant to the choice of reflections for refinement. *R*-factors based on *F*^2^ are statistically about twice as large as those based on *F*, and *R*- factors based on ALL data will be even larger.

### Fractional atomic coordinates and isotropic or equivalent isotropic displacement parameters (Å^2^)

**Table:**

	*x*	*y*	*z*	*U*_iso_*/*U*_eq_	
C1	0.17131 (13)	0.84678 (19)	0.25979 (4)	0.0187 (2)	
C2	0.09703 (13)	0.7573 (2)	0.29780 (4)	0.0211 (3)	
H2	0.0487	0.6188	0.2934	0.025*	
C3	0.09548 (13)	0.8752 (2)	0.34216 (4)	0.0226 (3)	
C4	0.16617 (13)	1.0821 (2)	0.34766 (4)	0.0234 (3)	
H4	0.1620	1.1628	0.3778	0.028*	
C5	0.24143 (13)	1.1703 (2)	0.31029 (4)	0.0214 (3)	
H5	0.2894	1.3090	0.3148	0.026*	
C6	0.24560 (12)	1.05129 (18)	0.26567 (4)	0.0182 (2)	
C7	0.31598 (12)	1.08697 (18)	0.22066 (4)	0.0183 (2)	
C8	0.41054 (13)	1.25324 (19)	0.20595 (4)	0.0211 (3)	
H8	0.4326	1.3796	0.2262	0.025*	
C9	0.47011 (13)	1.2281 (2)	0.16144 (4)	0.0224 (3)	
H9	0.5374	1.3364	0.1516	0.027*	
C10	0.43349 (13)	1.04460 (19)	0.12984 (4)	0.0203 (2)	
C11	0.33180 (13)	0.88417 (18)	0.14256 (4)	0.0185 (2)	
C12	0.27867 (12)	0.90402 (18)	0.18939 (4)	0.0181 (2)	
C13	0.01770 (15)	0.7822 (2)	0.38419 (5)	0.0301 (3)	
H13A	−0.0933	0.8209	0.3784	0.045*	
H13B	0.0674	0.8413	0.4170	0.045*	
H13C	0.0284	0.6239	0.3846	0.045*	
C14	0.50985 (13)	1.0114 (2)	0.08483 (4)	0.0237 (3)	
C15	0.47085 (14)	0.7998 (2)	0.05735 (4)	0.0243 (3)	
H15A	0.5436	0.6861	0.0731	0.029*	
H15B	0.4857	0.8159	0.0214	0.029*	
C16	0.30334 (14)	0.72833 (19)	0.05911 (4)	0.0208 (2)	
C17	0.27377 (17)	0.5034 (2)	0.03665 (5)	0.0290 (3)	
H17A	0.3465	0.4003	0.0558	0.043*	
H17B	0.2895	0.5048	0.0010	0.043*	
H17C	0.1662	0.4598	0.0386	0.043*	
C18	0.18336 (14)	0.8888 (2)	0.03275 (4)	0.0229 (3)	
H18A	0.0779	0.8353	0.0345	0.034*	
H18B	0.1966	0.9043	−0.0030	0.034*	
H18C	0.1983	1.0300	0.0497	0.034*	
N1	0.19001 (11)	0.76108 (16)	0.21306 (4)	0.0199 (2)	
H1	0.1519	0.6366	0.2005	0.024*	
O1	0.28283 (10)	0.70934 (13)	0.11287 (3)	0.0218 (2)	
O2	0.60210 (11)	1.14169 (18)	0.07137 (3)	0.0341 (2)	

### Atomic displacement parameters (Å^2^)

**Table:**

	*U*^11^	*U*^22^	*U*^33^	*U*^12^	*U*^13^	*U*^23^
C1	0.0168 (5)	0.0229 (6)	0.0158 (5)	0.0018 (4)	0.0006 (4)	−0.0007 (4)
C2	0.0178 (5)	0.0256 (6)	0.0194 (6)	−0.0015 (4)	0.0019 (4)	0.0010 (5)
C3	0.0165 (5)	0.0332 (7)	0.0179 (6)	0.0040 (5)	0.0020 (4)	0.0021 (5)
C4	0.0209 (6)	0.0308 (7)	0.0178 (6)	0.0048 (5)	0.0005 (4)	−0.0051 (5)
C5	0.0198 (5)	0.0224 (6)	0.0208 (6)	0.0029 (4)	−0.0008 (4)	−0.0027 (5)
C6	0.0156 (5)	0.0204 (6)	0.0176 (5)	0.0021 (4)	−0.0007 (4)	0.0005 (4)
C7	0.0169 (5)	0.0203 (6)	0.0165 (5)	0.0018 (4)	−0.0008 (4)	0.0001 (4)
C8	0.0214 (6)	0.0205 (6)	0.0197 (6)	−0.0028 (4)	−0.0018 (4)	0.0002 (4)
C9	0.0203 (5)	0.0247 (6)	0.0210 (6)	−0.0045 (5)	−0.0009 (4)	0.0041 (5)
C10	0.0180 (5)	0.0246 (6)	0.0175 (5)	0.0004 (4)	0.0006 (4)	0.0031 (4)
C11	0.0200 (5)	0.0183 (5)	0.0166 (5)	0.0019 (4)	0.0011 (4)	0.0008 (4)
C12	0.0171 (5)	0.0189 (5)	0.0177 (5)	0.0003 (4)	0.0008 (4)	0.0018 (4)
C13	0.0265 (6)	0.0436 (8)	0.0214 (6)	−0.0001 (6)	0.0075 (5)	0.0010 (5)
C14	0.0189 (5)	0.0342 (7)	0.0174 (6)	0.0005 (5)	0.0006 (4)	0.0054 (5)
C15	0.0247 (6)	0.0308 (7)	0.0185 (6)	0.0064 (5)	0.0067 (5)	0.0027 (5)
C16	0.0276 (6)	0.0210 (6)	0.0147 (5)	0.0014 (5)	0.0061 (4)	−0.0001 (4)
C17	0.0454 (8)	0.0219 (6)	0.0214 (6)	0.0018 (5)	0.0107 (5)	−0.0023 (5)
C18	0.0246 (6)	0.0238 (6)	0.0195 (6)	0.0017 (5)	0.0014 (4)	−0.0012 (5)
N1	0.0239 (5)	0.0189 (5)	0.0170 (5)	−0.0037 (4)	0.0043 (4)	−0.0015 (4)
O1	0.0317 (4)	0.0192 (4)	0.0156 (4)	−0.0020 (3)	0.0069 (3)	−0.0008 (3)
O2	0.0287 (5)	0.0506 (6)	0.0240 (5)	−0.0135 (4)	0.0072 (4)	0.0010 (4)

### Geometric parameters (Å, °)

**Table:**

C1—N1	1.3845 (14)	C11—C12	1.4010 (15)
C1—C2	1.3959 (16)	C12—N1	1.3847 (14)
C1—C6	1.4132 (16)	C13—H13A	0.9800
C2—C3	1.3887 (17)	C13—H13B	0.9800
C2—H2	0.9500	C13—H13C	0.9800
C3—C4	1.4123 (18)	C14—O2	1.2263 (15)
C3—C13	1.5079 (17)	C14—C15	1.5078 (18)
C4—C5	1.3843 (17)	C15—C16	1.5255 (17)
C4—H4	0.9500	C15—H15A	0.9900
C5—C6	1.4013 (16)	C15—H15B	0.9900
C5—H5	0.9500	C16—O1	1.4744 (13)
C6—C7	1.4447 (15)	C16—C17	1.5155 (17)
C7—C8	1.4064 (16)	C16—C18	1.5239 (16)
C7—C12	1.4086 (16)	C17—H17A	0.9800
C8—C9	1.3739 (17)	C17—H17B	0.9800
C8—H8	0.9500	C17—H17C	0.9800
C9—C10	1.4156 (17)	C18—H18A	0.9800
C9—H9	0.9500	C18—H18B	0.9800
C10—C11	1.4016 (16)	C18—H18C	0.9800
C10—C14	1.4718 (16)	N1—H1	0.8800
C11—O1	1.3638 (14)		
			
N1—C1—C2	129.29 (11)	C3—C13—H13B	109.5
N1—C1—C6	108.89 (10)	H13A—C13—H13B	109.5
C2—C1—C6	121.80 (10)	C3—C13—H13C	109.5
C3—C2—C1	118.47 (11)	H13A—C13—H13C	109.5
C3—C2—H2	120.8	H13B—C13—H13C	109.5
C1—C2—H2	120.8	O2—C14—C10	123.03 (12)
C2—C3—C4	119.90 (11)	O2—C14—C15	122.10 (11)
C2—C3—C13	119.81 (12)	C10—C14—C15	114.83 (10)
C4—C3—C13	120.28 (11)	C14—C15—C16	112.12 (10)
C5—C4—C3	121.77 (11)	C14—C15—H15A	109.2
C5—C4—H4	119.1	C16—C15—H15A	109.2
C3—C4—H4	119.1	C14—C15—H15B	109.2
C4—C5—C6	118.78 (11)	C16—C15—H15B	109.2
C4—C5—H5	120.6	H15A—C15—H15B	107.9
C6—C5—H5	120.6	O1—C16—C17	105.74 (9)
C5—C6—C1	119.24 (11)	O1—C16—C18	108.67 (9)
C5—C6—C7	133.95 (11)	C17—C16—C18	110.60 (10)
C1—C6—C7	106.79 (10)	O1—C16—C15	108.32 (9)
C8—C7—C12	120.57 (10)	C17—C16—C15	110.77 (10)
C8—C7—C6	133.09 (11)	C18—C16—C15	112.48 (10)
C12—C7—C6	106.29 (10)	C16—C17—H17A	109.5
C9—C8—C7	118.24 (11)	C16—C17—H17B	109.5
C9—C8—H8	120.9	H17A—C17—H17B	109.5
C7—C8—H8	120.9	C16—C17—H17C	109.5
C8—C9—C10	121.51 (11)	H17A—C17—H17C	109.5
C8—C9—H9	119.2	H17B—C17—H17C	109.5
C10—C9—H9	119.2	C16—C18—H18A	109.5
C11—C10—C9	120.74 (11)	C16—C18—H18B	109.5
C11—C10—C14	118.66 (11)	H18A—C18—H18B	109.5
C9—C10—C14	120.47 (11)	C16—C18—H18C	109.5
O1—C11—C12	117.93 (10)	H18A—C18—H18C	109.5
O1—C11—C10	124.60 (10)	H18B—C18—H18C	109.5
C12—C11—C10	117.46 (10)	C1—N1—C12	108.55 (9)
N1—C12—C11	129.31 (10)	C1—N1—H1	125.7
N1—C12—C7	109.45 (10)	C12—N1—H1	125.7
C11—C12—C7	121.24 (10)	C11—O1—C16	115.16 (9)
C3—C13—H13A	109.5		
			
N1—C1—C2—C3	179.15 (11)	O1—C11—C12—N1	5.46 (17)
C6—C1—C2—C3	0.73 (17)	C10—C11—C12—N1	−173.73 (11)
C1—C2—C3—C4	0.88 (17)	O1—C11—C12—C7	−175.89 (10)
C1—C2—C3—C13	−179.36 (10)	C10—C11—C12—C7	4.92 (16)
C2—C3—C4—C5	−1.64 (17)	C8—C7—C12—N1	177.69 (10)
C13—C3—C4—C5	178.61 (11)	C6—C7—C12—N1	0.03 (12)
C3—C4—C5—C6	0.72 (17)	C8—C7—C12—C11	−1.21 (16)
C4—C5—C6—C1	0.88 (16)	C6—C7—C12—C11	−178.86 (10)
C4—C5—C6—C7	−177.44 (11)	C11—C10—C14—O2	−178.92 (11)
N1—C1—C6—C5	179.66 (10)	C9—C10—C14—O2	−3.00 (18)
C2—C1—C6—C5	−1.63 (16)	C11—C10—C14—C15	−1.16 (15)
N1—C1—C6—C7	−1.60 (12)	C9—C10—C14—C15	174.76 (10)
C2—C1—C6—C7	177.11 (10)	O2—C14—C15—C16	−148.09 (12)
C5—C6—C7—C8	2.2 (2)	C10—C14—C15—C16	34.13 (14)
C1—C6—C7—C8	−176.28 (12)	C14—C15—C16—O1	−57.83 (12)
C5—C6—C7—C12	179.42 (12)	C14—C15—C16—C17	−173.36 (10)
C1—C6—C7—C12	0.95 (12)	C14—C15—C16—C18	62.29 (13)
C12—C7—C8—C9	−2.51 (16)	C2—C1—N1—C12	−176.93 (11)
C6—C7—C8—C9	174.40 (11)	C6—C1—N1—C12	1.64 (12)
C7—C8—C9—C10	2.39 (17)	C11—C12—N1—C1	177.74 (11)
C8—C9—C10—C11	1.44 (17)	C7—C12—N1—C1	−1.03 (12)
C8—C9—C10—C14	−174.39 (11)	C12—C11—O1—C16	162.98 (10)
C9—C10—C11—O1	175.84 (10)	C10—C11—O1—C16	−17.90 (15)
C14—C10—C11—O1	−8.26 (17)	C17—C16—O1—C11	168.56 (10)
C9—C10—C11—C12	−5.03 (16)	C18—C16—O1—C11	−72.69 (12)
C14—C10—C11—C12	170.87 (10)	C15—C16—O1—C11	49.79 (12)

### Hydrogen-bond geometry (Å, °)

**Table:**

*D*—H···*A*	*D*—H	H···*A*	*D*···*A*	*D*—H···*A*
C15—H15B···O2^i^	0.99	2.48	3.4034 (13)	155
N1—H1···Cg2^ii^	0.88	2.97	3.5822 (11)	128
C2—H2···Cg1^ii^	0.95	2.74	3.5332 (13)	141
C8—H8···Cg1^iii^	0.95	2.91	3.4124 (12)	114
C9—H9···Cg2^iii^	0.95	2.74	3.4372 (13)	130

Symmetry codes: (i) −*x*+1, −*y*+2, −*z*; (ii) −*x*, *y*−1/2, −*z*+1/2; (iii) −*x*+1, *y*+1/2, −*z*+1/2.
